# Isolinderalactone Induces Apoptosis, Autophagy, Cell Cycle Arrest and MAPK Activation through ROS–Mediated Signaling in Colorectal Cancer Cell Lines

**DOI:** 10.3390/ijms241814246

**Published:** 2023-09-18

**Authors:** Jith-Shyan Chen, Sheng-Chun Chiu, Sung-Ying Huang, Shu-Fang Chang, Kuan-Fu Liao

**Affiliations:** 1 Department of Obstetrics and Gynecology, Taichung Tzu Chi Hospital, Buddhist Tzu Chi Medical Foundation, Taichung 427213, Taiwan; cjschen.cjs@tzuchi.com.tw; 2 Department of Research, Taichung Tzu Chi Hospital, Buddhist Tzu Chi Medical Foundation, Taichung 427213, Taiwan; honeyhopes@gmail.com (S.-C.C.); fantac10@gmail.com (S.-F.C.); 3 Department of Laboratory Medicine, Taichung Tzu Chi Hospital, Buddhist Tzu Chi Medical Foundation, Taichung 427213, Taiwan; 4 General Education Center, Tzu Chi University of Science and Technology, Hualien 973302, Taiwan; 5 Department of Ophthalmology, Hsinchu Mackay Memorial Hospital, Hsinchu 300044, Taiwan; hopes929@gmail.com; 6 Department of Internal Medicine, Taichung Tzu Chi Hospital, Buddhist Tzu Chi Medical Foundation, Taichung 427213, Taiwan

**Keywords:** colorectal cancer, isolinderalactone, apoptosis, autophagy, G2/M arrest, ROS

## Abstract

Colorectal cancer (CRC) is one of the most common malignancies worldwide. Isolinderalactone (ILL), a sesquiterpene isolated from the root extract of *Lindera aggregata*, has been reported to exhibit anti–proliferative and anti–metastatic activities in various cancer cell lines. However, the mechanisms associated with its antitumor effects on CRC cells remain unclear. ILL treatment significantly suppressed proliferation and induced cell cycle G2/M arrest in CRC cells by inhibiting the expression of cyclin B, p–cdc2, and p–cdc25c and up–regulating the expression of p21. In addition, ILL induced mitochondria–associated apoptosis through the up–regulation of cleaved –caspase–9 and –3 expression. ILL induced autophagy by increasing the levels of LC3B in CRC cells, which was partially rescued by treatment with an autophagy inhibitor (chloroquine). Furthermore, ILL increases the accumulation of reactive oxygen species (ROS) and activates the MAPK pathway. Application of the ROS scavenger, N–acetyl cysteine (NAC), effectively inhibited ILL toxicity and reversed ILL–induced apoptosis, cell cycle arrest, autophagy, and ERK activation. Taken together, these results suggest that ILL induces G2/M phase arrest, apoptosis, and autophagy and activates the MAPK pathway via ROS–mediated signaling in human CRC cells.

## 1. Introduction

Colorectal cancer (CRC) is the third most commonly diagnosed malignancy and the second leading cause of cancer–related death worldwide [1]. More than 1.9 million newly diagnosed cases and approximately 0.9 million CRC–related deaths were recorded in 2020 [2]. CRC is treatable in the early stages, mainly by surgical resection, whereas chemotherapy is the primary treatment option for patients in advanced stages. The commonly used chemotherapy drugs for CRC include 5–fluorouracil, oxaliplatin, and irinotecan. However, the overall 5–year survival rate of patients with CRC remains low, owing to the limitations of these regimens, such as their severe adverse effects, low response rates, and drug resistance. Therefore, the development of new, effective, and safe therapeutic agents for the treatment of CRC is needed.

Reactive oxygen species (ROS) play key roles in regulating cell proliferation and death in several types of cancer, including CRC [3]. Overproduction of ROS leads to oxidative stress, which can cause significant cytotoxicity via apoptosis, autophagy, or necrosis [4]. In addition, ROS have been reported to suppress CRC growth through the activation of mitogen–activated protein kinase (MAPK) signaling cascades [5]. Furthermore, excessive ROS accumulation induces cell cycle arrest and regulates autophagy during CRC treatment [6]. Although the role of autophagy in cancer therapy remains controversial, targeting and modulating autophagy is an alternative strategy for cancer treatment [7,8]. Therefore, targeting ROS–mediated signaling pathways may be beneficial for the treatment of CRC.

Natural products and their derivatives are among the most productive sources for drug discovery [9]. *Lindera aggregata*, also known as Wu Yao, is a traditional medicine widely used in Asian countries to treat abdominal pain and rheumatic diseases [10]. Linderalactone, linderane, and isolinderalactone (ILL) are the main sesquiterpenes isolated from the root extracts of *L. aggregata*. Among these three derivatives, ILL has been reported to exhibit greater anti–proliferative and anti–metastatic activities in various cancer cells [11,12,13]. ILL has been shown to inhibit proliferation and induce cell cycle arrest through the regulation of the Fas/Fas–ligand–mediated pathway in non–small human lung cancer A549 cells [11]. In addition, Hwang et al. demonstrated the inhibitory effects of ILL on the expression of X–linked inhibitor of apoptosis protein (XIAP), BCL–2, and survivin, resulting in activation of the apoptosis pathway in glioma cells [12]. Furthermore, a recent report revealed that ILL induces the death of human ovarian cancer cells by up–regulating mitochondrial superoxide and inactivating the STAT3–mediated pathway [14]. However, the anticancer effects of ILL on CRC remain unclear and require further investigation. In this study, we aimed to investigate the anti–cancer effects of ILL on CRC and elucidate the underlying mechanisms using human HCT15 and HCT116 cells. Our results suggested that ILL induces apoptosis, cell cycle G2/M arrest, and autophagy by activating the MAPK pathway via ROS–mediated signaling in human CRC cells.

## 2. Results

### 2.1. ILL Inhibited the Proliferation of Human CRC Cells

**Figure 1:**
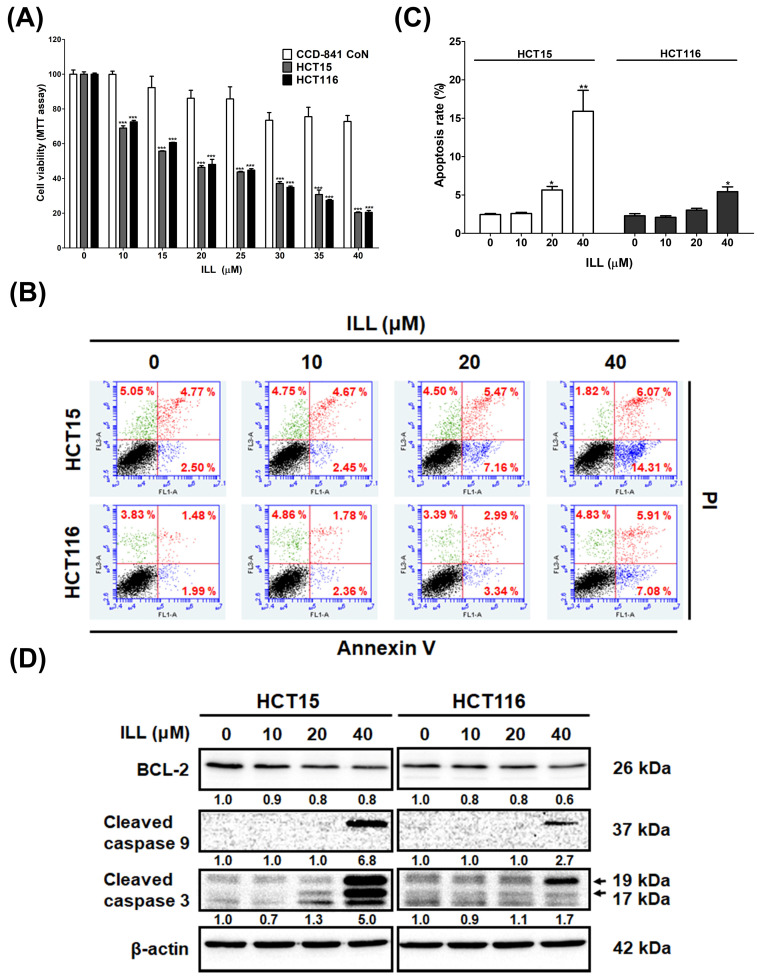
ILL treatment inhibits proliferation and induces apoptosis in CRC cells. (**A**) Human CRC cells and normal human colon epithelial cell line CCD 841 CoN were treated with vehicle control (0.1% DMSO) or different concentrations of ILL (0–40 μM) for 24 h, and cell viability was determined using MTT assay. Data are presented as the mean ± SD from three different experiments. *** *p* < 0.001 versus control. (**B**) Cells were treated with 0–40 μM of ILL for 24 h and then subjected to annexin V–FITC staining followed by flow cytometry analysis. (**C**) The percentage of apoptotic cells in each group is shown by histograms. Data are presented as the mean ± SD from three different experiments. * *p* < 0.05 versus control and ** *p* < 0.01 versus control. (**D**) Cells were treated with different concentrations of ILL (0–40 μM) for 24 h and subjected to Western blot analysis with antibodies against BCL–2, cleaved caspase–9, and cleaved caspase–3.

The cytotoxic effect of ILL on CRC cells was examined. Human HCT15 and HCT116 CRC cells with a normal human colon epithelial cell line, CCD 841 CoN, were incubated with different concentrations (10–40 μM) of ILL for 24 h. MTT assays revealed that the proliferation of CRC cells was significantly suppressed in a dose–dependent manner after ILL treatment (Figure 1A and Figure S1). The IC_50_ levels for HCT15 and HCT116 at 24 h were 22.1 ± 0.3 and 22.4 ± 0.1 μM, respectively. However, the viability of CCD 841 CoN cells decreased by only 20%, suggesting that ILL–induced cytotoxicity was selective for malignant rather than normal colorectal epithelial cells. To explore whether ILL inhibited the proliferation of CRC cells by inducing apoptosis, apoptotic cells were detected by annexinV–PI staining, followed by a flow cytometry analysis (Figure 1B). Treatment with increasing concentrations of ILL significantly increased the percentage of apoptotic cells in a dose–dependent manner. The percentage of apoptotic cells after 40 μM of ILL treatment was increased by 15.9 ± 6.7% (HCT15) and 5.4 ± 1.6% (HCT116) compared to the control group (Figure 1C). In addition, ILL treatment increased the levels of apoptosis–related proteins such as cleaved caspase–9 and –3 in CRC cells (Figure 1D). Taken together, these results suggest that ILL treatment inhibits cell proliferation and induces apoptosis in human CRC cells.

### 2.2. Transcriptome Analysis of ILL–Treated Human CRC Cells

**Figure 2:**
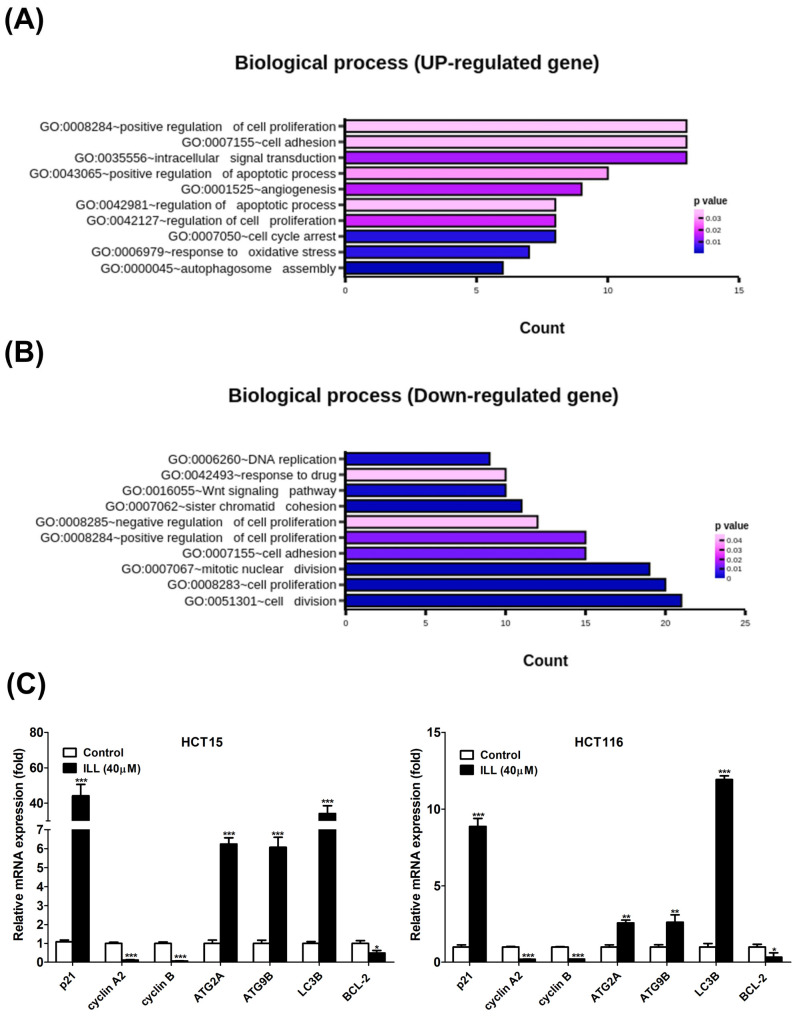
Effects of ILL on human CRC cells. Cells were treated with 40 μM of ILL followed by RNA–seq analysis. The (**A**) up–regulated biological process and the (**B**) down–regulated biological process that were enriched after ILL –treatment are shown. (**C**) Cells were treated with 40 μM of ILL for 24 h, and the up–regulated genes related to autophagy induction and the down–regulated genes related to cell cycle progression were validated using real–time PCR. Data are presented as the mean ± SD from three different experiments. * *p* < 0.05 versus control, ** *p* < 0.01 versus control, and *** *p* < 0.001 versus control.

To clarify how ILL inhibits cell proliferation and induces cell apoptosis in human CRC cells, we treated HCT15 cells with 40 μM of ILL for 24 h and then subjected them to RNA sequencing (RNA–seq) analysis to identify the candidate genes induced by ILL treatment. The RNA–seq analysis yielded remarkable results, revealing 998 significantly up–regulated and 627 significantly down–regulated differential–ly expressed genes (DEGs) in ILL–treated cells compared to control cells. To unravel the biological processes associated with ILL–treated cells, we conducted an in–depth investigation of these DEGs using Gene Ontology (GO) enrichment analysis. Remarkably, our investigation revealed 10 enriched biological processes primarily driven by up–regulated genes and another 10 enriched processes attributed to down–regulated genes in ILL–treated cells (Figure 2A,B). Notably, the enriched up–regulated processes in ILL–treated cells primarily encompassed key aspects of cell proliferation regulation, apoptotic processes, and the response to oxidative stress (e.g., autophagosome assembly). In contrast, the enriched down–regulated processes appeared to be predominantly associated with cell cycle regulation (e.g., mitotic nuclear division and cell division). To extend our exploration to uncover the potential biological pathways affected by ILL treatment, we used DAVID software to perform KEGG pathway analysis of the DEGs derived from the comparison between ILL–treated and control cells. Our analysis revealed seven enriched pathways in the up–regulated DEGs and another seven pathways enriched in the down–regulated DEGs in ILL–treated cells (Figure S2). Among these pathways, the up–regulation of autophagy and the down–regulation of the cell cycle were consistent with the rest of our experimental results (Figure 2C). Real–time PCR verified that some of the representative genes of autophagy induction (ATG2A, ATG9B, and LC3) were up–regulated, and those of the cell cycle (cyclin A2 and cyclin B) were down–regulated after ILL treatment of CRC cells for 24 h. These data suggest that autophagy induction and cell cycle inhibition following ILL treatment may play a role in ILL–mediated anti–proliferative effects in CRC cells.

### 2.3. ILL Induced Cell Cycle G2/M Arrest in Human CRC Cells

**Figure 3:**
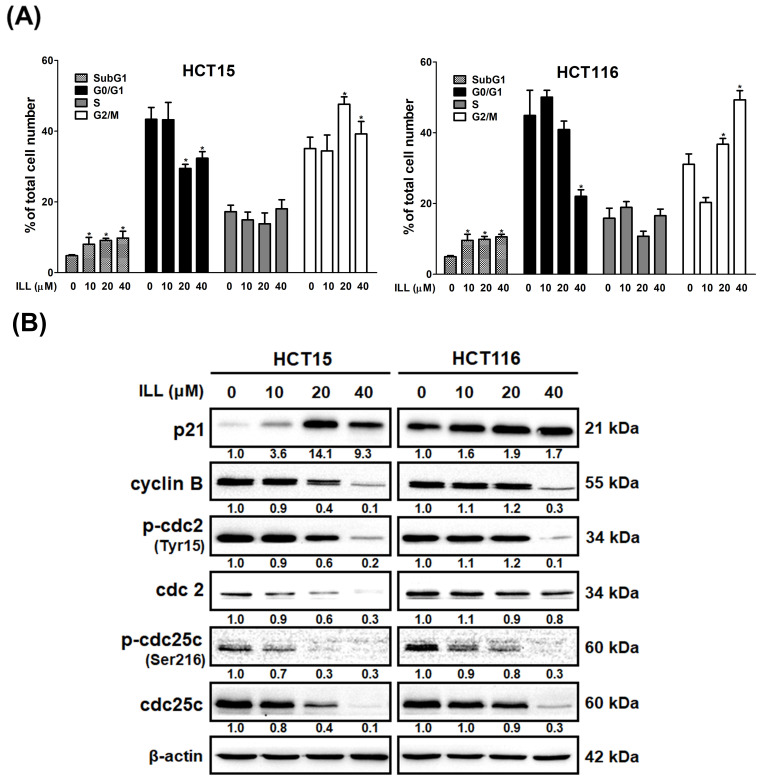
ILL induces G2/M phase arrest in CRC cells. (**A**) Cells were treated with 0–40 μM of ILL for 24 h, and the cell cycle distributions were then analyzed by flow cytometry. Data are presented as the mean ± SD from three different experiments. * *p* < 0.05 versus control. (**B**) Cells were treated with 0–40 μM of ILL for 24 h, the expression levels of p21, cyclin B, p–cdc2 (Tyr15), cdc2, p–cdc25c (Ser216), and cdc25c were determined by Western blot analysis.

To determine the role of cell cycle arrest in ILL–induced proliferation inhibition, we examined the cell cycle progression of ILL–treated CRC cells by flow cytometry. As shown in Figure 3A and Figure S3, the proportion of cells that accumulated in the G2/M phase increased after ILL treatment, and this was accompanied by a decrease in the percentage of cells in the G0/G1 phase. In addition, ILL decreased the expression levels of cyclin B, p–cdc2 (Tyr15), cdc2, p–cdc25c (Ser216), and cdc25c but increased the levels of p21 in a dose–dependent manner (Figure 3B). Taken together, these results demonstrate that ILL suppressed the proliferation of CRC cells by inducing cell cycle arrest in the G2/M phase.

### 2.4. ILL Stimulated Autophagy in Human CRC Cells

**Figure 4:**
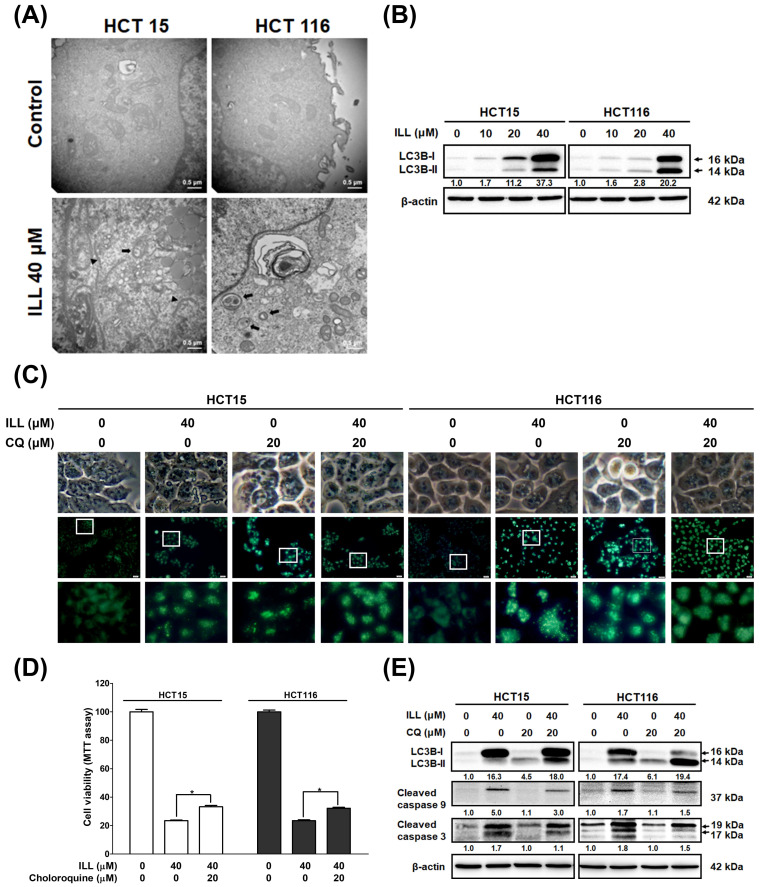
Effects of ILL on the activation of autophagy in CRC cells. (**A**) Representative TEM micrographs were acquired from CRC cells treated with or without 40 μM of ILL for 24 h. Autophagosomes (black arrow), secondary lysosome (white arrow), and phagophores (arrowhead) were more prominently observed in ILL–exposed cells than control cells. (**B**) Cells were treated with various concentrations of ILL for 24 h and then subjected to Western blot analysis with antibodies against LC3B–I and LC3B–II. (**C**) Cells were treated with 0.5% DMSO (control) or 40 μM of ILL in the presence or absence of the autophagy inhibitor chloroquine (CQ; 20 μM) for 24 h and then subjected to MDC staining (green) for autophagy detection. (**D**) Cells were pretreated with CQ for 2 h and then treated with ILL for 24 h. Cell viability was determined using an MTT assay. Data are presented as the mean ± SD from three different experiments. * *p* < 0.05 versus control. (**E**) Cells were pretreated with CQ for 2 h and then treated with ILL for 24 h, after which the levels of LC3B–I, LC3B–II, and cleaved caspase–9 and –3 were analyzed by Western blot analysis.

Autophagy plays a key role in modulating cell survival and death, and it has been implicated in the development and progression of various diseases [15]. The results of RNA–seq analysis after ILL treatment also suggested that autophagy induction may be involved in the anti–cancer effects of ILL on CRC cells (Figure 2). To clarify whether ILL could induce autophagy in CRC cells, transmission electron microscopy (TEM) was used to analyze intracellular morphological changes in CRC cells after treatment with ILL for 24 h (Figure 4A and Figure S4). Cells with intact intracellular organelles, including the mitochondria, nuclei, and nuclear membranes were observed in the vehicle control. In contrast, a marked increase in vacuolar structures was observed in ILL–treated cells. Consistently, treatment with ILL induced the expression of autophagy–related proteins LC3B–I and LC3B–II in a dose–dependent manner (Figure 4B). These results indicate that ILL promoted autophagy in CRC cells.

To further elucidate the relationship between autophagy and apoptosis, we used the autophagy inhibitor chloroquine (CQ) to block autophagy and determine the effect of ILL on cell death. As shown in Figure 4C, monodansylcadaverine (MDC) staining revealed that ILL treatment significantly induced the number of green puncta, whereas no difference was observed regardless of the presence of CQ (Figures S5 and S6). This result could be attributed to the fact that CQ exerts its inhibitory effect on autophagy by impairing the fusion of autophagosomes with lysosomes. As a result, the accumulation of autophagosomes did not show a significant change in the number of green puncta after MDC staining. Western blot analysis demonstrated a significant increase in LC3B expression levels in CRC cells following ILL treatment, which is consistent with the results observed by MDC staining (Figure 4E). However, the addition of CQ did not significantly inhibit the expression of LC3B. Furthermore, the expression levels of LC3B–II were slightly increased in the CQ group, either with or without ILL treatment, indicating the accumulation of autophagosomes after CQ treatment. The MTT assay revealed that the viability of CRC cells was increased in the CQ–treated group compared with the ILL–alone group (from HCT15: 24.3 ± 1.9% to 32.6 ± 2.4%, and from HCT116: 23.4 ± 0.9% to 32.2 ± 1%, respectively), suggesting that the inhibition of autophagy by CQ partially attenuated the autophagic cell death induced by ILL treatment (Figure 4D). Western blot analysis also showed that CQ treatment slightly decreased the levels of cleaved caspase–9 and –3 in the presence of 40 μM of ILL (Figure 4E). Collectively, these results indicate that autophagy positively mediates apoptosis in ILL–treated CRC cells.

### 2.5. Generation of ROS in Human CRC Cells after ILL Treatment

**Figure 5:**
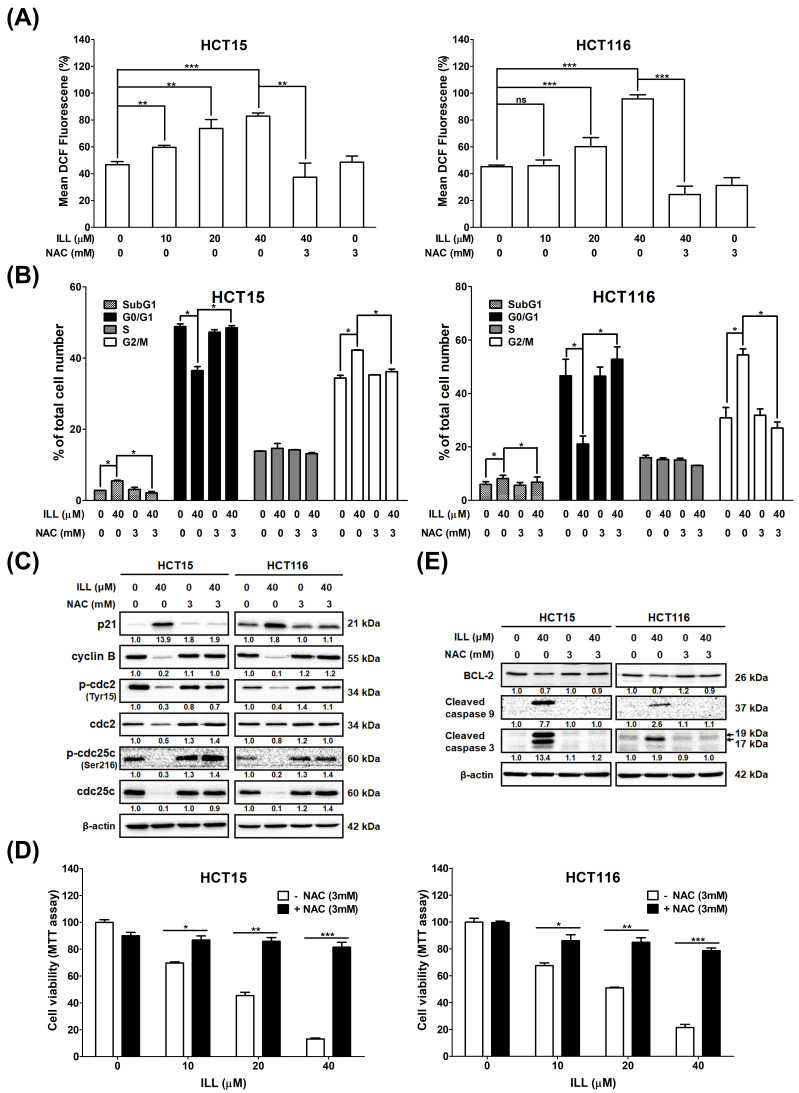
Effects of ILL on ROS generation in CRC cells. (**A**) Cells were treated with various concentration of ILL in the presence or absence of NAC for 24 h. The cells were then subjected to 10 μM DCFDA staining for 30 min, and the resulting fluorescent intensity was analyzed by flow cytometry. Data are presented as the mean ± SD from three different experiments. ** *p* < 0.01 versus control and *** *p* < 0.001 versus control, ns: not significant. (**B**) Cells were treated with or without 40 μM of ILL in the presence or absence of 3 mM of NAC for 24 h, and the cell cycle distributions were then analyzed by flow cytometry. Data are presented as the mean ± SD from three different experiments. * *p* < 0.05 versus control. (**C**) Cells were treated with or without 40 μM of ILL in the presence or absence of 3 mM of NAC for 24 h, and the levels of p21, cyclin B, p–cdc2 (Tyr15), cdc2, p–cdc25c (Ser216), and cdc25c was determined by Western blot analysis. (**D**) Cells were treated with various concentrations of ILL in the presence or absence of 3 mM of NAC for 24 h, and then they were subjected to cell viability analysis using the MTT assay. Data are presented as the mean ± SD from three different experiments. * *p* < 0.05 versus control, ** *p* < 0.01 versus control, and *** *p* < 0.001 versus control. (**E**) Cells were treated with or without 40 μM of ILL in the presence or absence of 3 mM of NAC for 24 h, and the levels of BCL–2 and cleaved caspase–9 and –3 were determined by Western blot analysis.

Reactive oxygen species (ROS), which are generated and accumulate in response to oxidative stress, are potential inducers of apoptosis, autophagy, and cell cycle arrest. To assess whether ILL induced oxidative stress in CRC cells, ROS generation was measured using the DCFDA staining, followed by flow cytometry analysis. As shown in Figure 5A and Figure S7, the ROS content of CRC cells was significantly increased by ILL treatment in a dose–dependent manner, as revealed by DCFDA staining. However, this increase was reversed by pretreatment with the ROS scavenger *N*–acetyl–L–cysteine (NAC). Treatment with 40 μM of ILL increased ROS content to 84.3 ± 2% (HCT15) and 95.7 ± 3% (HCT116) compared to the control group, while pretreatment with 3 mM of NAC significantly reduced the ROS content to 40.3 ± 8.9% (HCT15) and 24.4 ± 6.3% (HCT116), respectively.

### 2.6. ILL–Mediated ROS Generation Contributed to Apoptosis, Autophagy, and Cell Cycle Arrest

Because ILL induces ROS accumulation, which may be involved in proliferation inhibition on CRC cells, its relationship with the cell cycle, apoptosis, and autophagy was further investigated. Pretreatment with NAC significantly reduced the proportion of cells in the G2/M phase from 43.7 ± 2.8% to 35.6 ± 4.3% (HCT15) and from 58.1 ± 3.9% to 27.3 ± 3.2% (HCT116), respectively (Figure 5B). In addition, pretreatment with NAC increased the expression levels of G2/M phase regulatory proteins such as cyclin B, p–cdc2, and p–cdc25c, which were suppressed by ILL treatment (Figure 5C). These results suggest that pretreatment with NAC partially reversed the effects of ILL on the cell cycle. Furthermore, the MTT assay revealed that pretreatment with NAC reversed the inhibitory effect of ILL on the proliferation of CRC cells. Pretreatment with 3 mM of NAC significantly rescued the death of CRC cells from 13.1 to 81.5% (40 μM of ILL, HCT15) and from 21.5 to 78.7% (40 μM of ILL, HCT116; Figure 5D). Western blotting analysis revealed that pretreatment with 3 mM of NAC decreased the levels of cleaved caspase–9 and –3 apoptotic proteins induced by ILL treatment (Figure 5E). In summary, these results suggest that ILL treatment induced ROS accumulation, thus promoting the apoptosis and G2/M cell cycle arrest of CRC cells.

### 2.7. ILL Activated MAPK Signaling and Affected Apoptosis, Autophagy, and Cell Cycle Arrest

**Figure 6:**
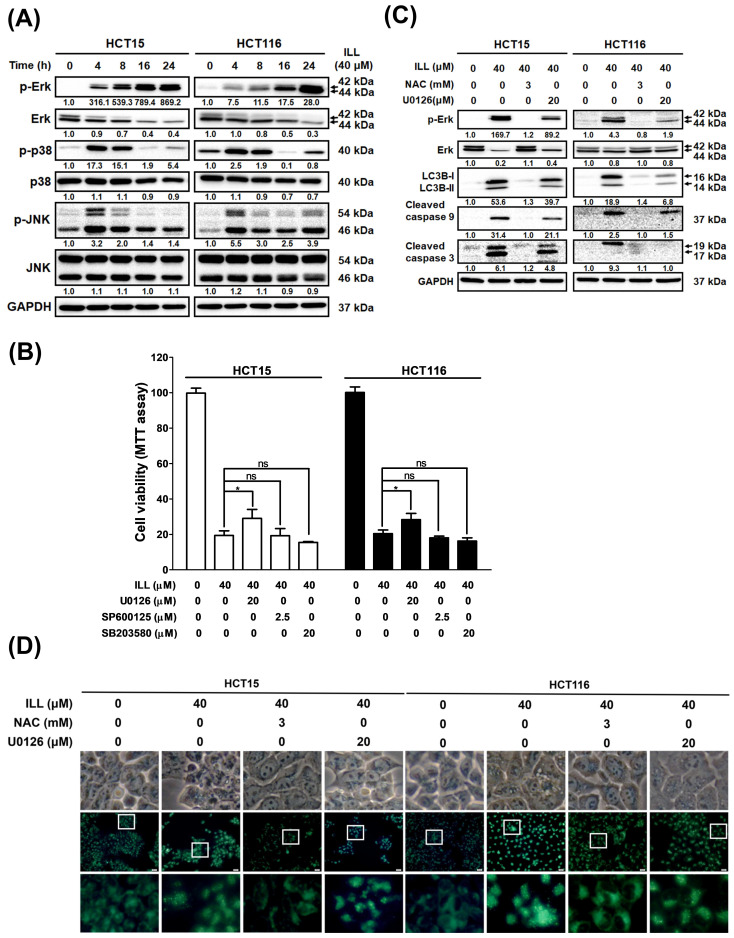
The ROS–mediated signaling pathway involved in the ILL–induced autophagy and apoptosis of CRC cells. (**A**) Cells were treated with 40 μM of ILL for the indicated time points and the levels of p–ERK, ERK, p–JNK, JNK, p–p38, and p38 were determined by Western blot. (**B**) Cells were pretreated with 20 μM of ERK inhibitor (U0126), 2.5 μM of JNK inhibitor (SP600125), or 20 μM of p38 inhibitor (SB203580) for 2 h, followed by treatment with 40 μM of ILL for 24 h. They were then subjected to an MTT assay for cell viability analysis. Data are presented as the mean ± SD from three different experiments. * *p* < 0.05 versus control, ns: not significant. (**C**) Cells were pretreated with 3 mM of NAC or 20 μM of ERK inhibitor (U0126) for 2 h, followed by treatment with 40 μM of ILL for 24 h. The levels of p–ERK, ERK, LC3B–I, LC3B–II, and cleaved caspase–9 and –3 were analyzed by Western blot. (**D**) Cells were pretreated with 3 mM of NAC or 20 Μm of ERK inhibitor (U0126) for 2 h, followed by treatment with 40 μM of ILL for 24 h. Cells were then subjected to MDC staining and the images were captured by fluorescence microscope.

Several reports have indicated that ROS play roles in apoptosis, autophagy, and cell cycle arrest by affecting the MAPK signaling pathway. To further elucidate whether ILL can activate the MAPK pathway by inducing ROS generation, CRC cells were treated with ILL for the indicated times, and the protein expression profile of members of the MAPK pathway was analyzed by Western blot (Figure 6A). Western blot revealed that the levels of phosphorylated p38 and JNK were transiently increased from 4 to 8 h after ILL treatment, regardless of whether the level of phosphorylated ERK was sustained from 4 to 24 h. In addition, cell viability in response to 40 μM of ILL treatment (19.4 ± 2.7%) was significantly increased by pre–treatment with an ERK inhibitor (U0126, 29.02 ± 5.1%), but not by pretreatment with p38 (SB203580, 19.3 ± 4%) or JNK inhibitors (SP600125, 15.5 ± 0.5%; Figure 6B). Consistently, the levels of apoptosis–related proteins, such as cleaved caspase–3 and –9, were suppressed in the presence of an ERK inhibitor (U0126) in ILL–treated CRC cells (Figure 6C). To further investigate the role of the ERK signaling pathway in ILL–induced autophagy, the ROS scavenger NAC and an ERK inhibitor (U0126) were used. MDC staining data showed that treatment with NAC significantly decreased the ILL–induced fluorescence intensity. However, treatment with the ERK inhibitor (U0126) only partially decreased the fluorescence intensity compared to the intensity in the NAC group (Figure 6D, Figures S8 and S9). Similarly, the expression levels of autophagy–related proteins LC3B–I and LC3B–II were partially suppressed in the presence of the ERK inhibitor (U0126) compared to their levels in the presence of NAC in ILL–treated CRC cells (Figure 6C). Furthermore, NAC treatment was more effective at blocking ERK phosphorylation than treatment with the ERK inhibitor (U0126; Figure 5C). Taken together, these results suggest that ROS may act upstream of ERK activation during ILL–induced apoptosis and autophagy in CRC cells.

## 3. Discussion

Current treatments for CRC include surgical resection, radiation therapy, chemotherapy, immunotherapy, and platinum–based chemotherapy. Although these therapies lead to CRC responses, most are eventually associated with the recurrence of chemo–resistance or serious side effects, such as neurological toxicities [16]. Thus, the development of more effective treatments for CRC is urgently required. ILL, an active ingredient of *L. aggregate*, has been shown to have anticancer activity against lung, ovarian, and pancreatic cancers. However, the molecular mechanisms underlying the anticancer effects of ILL on CRC remain unclear and require further investigation. In this study, we first demonstrated that ILL inhibits CRC cell proliferation but to a lesser extent in normal colon epithelial CCD 841 CoN cells (Figure 1). Our results showed that ILL induced mitochondria–associated apoptosis, G2/M cell cycle arrest, and autophagy in CRC cells. Moreover, the induction of ROS production by ILL resulted in ERK phosphorylation, leading to apoptosis, cell cycle arrest, and autophagy. Our study revealed that ILL induced cell cycle arrest at the G2/M phase via the down–regulation of cyclin B1, cdc2, and cdc25c, which may be the underlying mechanism responsible for the ILL–mediated anti–proliferative effect on CRC cells.

Autophagy is an important mechanism involved in maintaining homeostasis through the turnover of cellular components and the degradation of cellular materials in lysosomes [7]. Autophagy has both beneficial and detrimental effects in cancer therapy. Excessive autophagy contributes to autophagic cell death and exhibits cytoprotective effects that facilitate cancer cell survival under several cancer treatment conditions [17]. Recent reports indicated that resveratrol treatment increased autophagic flux, leading to the induction of autophagic cell death in A549 lung cancer cells [18]. Another study showed that LZ1, a peptide derived from snake venom cathelicidin, suppressed pancreatic cancer growth in vitro and in vivo by inducing autophagic cell death through activation of the AMPK pathway [19]. In our study, ILL–induced autophagy was revealed by an increased number of autophagic vacuoles observed using TEM imaging. The expression levels of autophagy–related proteins and mRNAs were consistently elevated after ILL treatment. Moreover, blocking autophagy with CQ reversed ILL–induced cell death and apoptosis–related gene expression, indicating that ILL–induced autophagy may promote the death of CRC cells. To our knowledge, this is the first study to demonstrate that ILL induces cell death upon autophagy induction.

Intracellular ROS acts as a double–edged sword in tumor initiation, progression, and treatment [20,21]. Overproduction of ROS is involved in the pathogenesis of several chronic diseases, including cancer. However, there is accumulating evidence that excessive ROS production induces oxidative stress, which triggers apoptotic cell death in cancer cells, including CRC cells [22]. ROS also induces cell cycle arrest accompanied by increased oxidative stress, resulting in increased cytotoxicity and cell death in CRC cells [23]. In this study, our data revealed that ILL stimulated ROS accumulation, which led to apoptosis and autophagy–related CRC cell death, which is consistent with a previous report [23]. Our results demonstrated that ILL treatment increased ROS generation, leading to mitochondria–associated apoptotic cell death. In addition, pretreatment with the ROS scavenger NAC significantly attenuated ILL–induced ROS production, blocked G2/M cell cycle arrest, and restored cell viability. Furthermore, the induction of autophagy was attenuated and the expression of autophagy–related LC3–I/II was diminished by NAC treatment in ILL–treated CRC cells, suggesting that ILL–induced autophagy is dependent on ROS production. Hydrogen peroxide (H_2_O_2_) and 2–methoxyestradiol (2–ME) induced autophagic cell death in various cancer cells, which was inhibited by 3–MA or the deletion of *Beclin–1* or *Atg7* but not by pan caspase inhibitor Z–VAD [24,25]. Blocking autophagy did not reduce ROS generation, which is consistent with our results and places ROS upstream of autophagy. Recently, several reports indicated that anticancer agents induced ROS production followed by activation of autophagy and induction of autophagic cell death in oral squamous cell carcinoma, prostate cancer, and CRC cells [26,27,28]. However, the exact mechanism by which autophagy achieves this outcome remains unclear. Zhao et al. reported that excessive oxidative stress leads to acetylation of FoxO1 by inducing its dissociation from sirtuin–2 (SIRT2) in HCT116 cells. The acetylated FoxO1 binds to *Atg7* in the cytosol, leading to the induction of autophagy, autophagic cell death, and tumor suppression in vitro and in vivo [29]. Transplantation of FoxO1–expressing cancer cells after stable knockdown of *Atg7* showed no tumor suppressive effect, demonstrating that FoxO1 exerts tumor suppressor activity by inducing autophagic cell death. Consistently, our RNA–seq results showed that ILL treatment up–regulated the FoxO signaling pathway (Figure S2). Furthermore, the RNA–seq data showed that ILL treatment induced the expression of FoxO1 (4.42–fold) and *Atg7* (2.37–fold) compared to the control group. Another study investigated how β–elemene, extracted from the genus Curcuma, affects CRC cells in vitro and in vivo. The results reported that β–elemene increased intracytoplasmic ROS levels, decreased the phosphorylation of mTOR, and led to autophagic cell death via activation of the ROS/AMPK/mTOR pathway [30]. Taken together, our results suggest that the anticancer mechanisms of ILL may involve the activation of ROS–mediated mitochondrial dysfunction and autophagy, leading to growth inhibition and the death of CRC cells.

MAPK signaling pathways regulate cell proliferation, differentiation, and apoptosis and are triggered in response to oxidative stress [31]. Excessive intracellular ROS has been reported to trigger cell apoptosis via the activation of the MAPK pathways, such as the p38 and JNK signaling cascades [21]. Here, we investigated whether ILL–induced ROS production activates MAPK pathways in CRC cells. ILL increased ROS generation (Figure 5A) and p–ERK, p–p38, and p–JNK protein levels (Figure 6A) in CRC cells. Co–treatment with an ERK inhibitor (U0126), but not with a p38 inhibitor (SB203580) or JNK inhibitor (SP600125), partially rescued ILL–induced cell death in CRC cells. In addition, co–treatment with an ERK inhibitor (U0126) partially reduced the expression levels of autophagy– and apoptosis–related proteins, which were dramatically abolished by pretreatment with the ROS scavenger NAC. Together, these results support the hypothesis that ILL induces apoptosis through the ERK pathway, which is mediated by excessive intracellular ROS production.

A recent report by Kwak and colleagues showed the effect of ILL on HCT116 cells, which has some discrepancies with our results [23]. In their report, 9 μM of ILL was used to study the effect on CRC cells. The ILL–induced apoptotic population was examined by annexin V–FITC staining (33.4%) and PI staining (39.6%), in contrast to our results using 40 μM of ILL (5.4% and 10.6%, respectively). Notably, their assessment of cell cycle distribution following 9 μM of ILL treatment showed only about 10% G2/M arrest, which differed from our observations using 40 μM of ILL (25.5%). Furthermore, they investigated the influence of ILL on autophagy using an LC3 antibody–based kit and Western blotting (data not shown). In their analysis, no significant changes in the LC3 intensity signal or LC3 protein expression were observed. However, in our study, we demonstrated autophagy induction after 40 μM of ILL treatment in CRC cells by TEM, MDC staining, and Western blotting. The differences in these experimental results may be attributed to the different doses of ILL used. Their report indicated that ILL treatment induced ROS–mediated apoptosis through the JNK/p38 pathway in CRC cells. Among the MAPKs, JNK and p38 are particularly involved in the induction of apoptosis following exposure of cells to various stress stimuli, whereas the ERK cascades mainly process signals stimulated by cell growth factors. Nevertheless, there are several examples showing that various insults can induce apoptosis through activation of the ERK signaling pathway. One study demonstrated that coplanar polychlorinated biphenyls (Co–PCBs) induced G2 cell cycle arrest and apoptosis through p38 and ERK activation in human extravillous cytotrophoblast–derived transformed cells [32]. Another study showed that DNA damage induced by cisplatin treatment in ovarian cancer A2780 cells activated ERK and increased p53 protein levels [33]. A recent report highlighted the contribution of ERK activation to the anti–proliferative and apoptotic effects of NSC 95397 (a quinone–based small molecule compound) in CRC cell lines [34]. It is well known that the pro– or anti–apoptotic effects of MAPK activation depend on the duration of the signal. Several reports have shown that transient activation of JNK and p38 promotes cell survival, whereas sustained activation is associated with the induction of apoptosis [35,36,37]. Furthermore, the persistence of ERK activation has been attributed to the presence of ROS during the cell death program, which is consistent with our findings [38]. In the present study, ILL induced sustained activation of ERK from 4 to 24 h, while transient activation of p38 and JNK was shown from 4 to 8 h (Figure 6A). However, Kwak and colleagues examined p38 and JNK activation but not ERK after 48 h of ILL treatment in CRC cells. Therefore, (1) the duration and timing of the MAPK assay and (2) the cell type and the stimuli may underlie the discrepancy in these results.

In a previous report, the body weight of mice was monitored during the entire 4 weeks of ILL treatment, and the data showed no differences between the groups in the brain tumor research [12]. In addition, no significant toxicity was observed in the lung, liver, and kidney of ILL–treated mice in a breast cancer study [13]. In the present study, the anticancer effects and molecular mechanisms of ILL were based on in vitro assays of human CRC cell lines, which limits the generalizability of the obtained results. Therefore, we will further validate the anticancer effects and underlying mechanisms of ILL on CRC through in vivo models such as the PDE system [39] or the zebrafish model [40].

**Figure 7:**
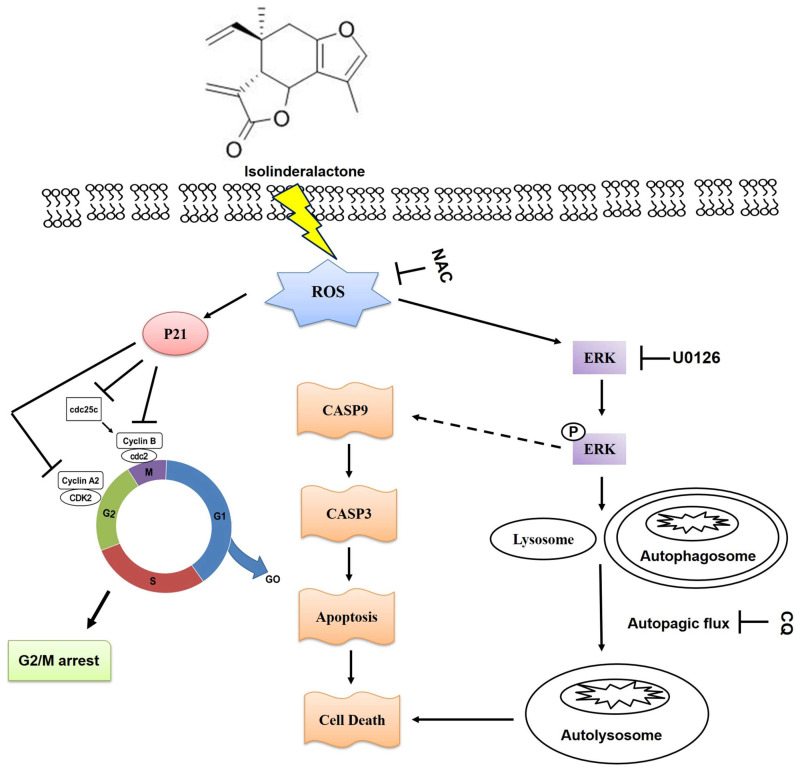
The proposed mechanism of ILL–induced apoptosis, cell cycle arrest, autophagy, and ERK activation through the ROS–mediated signaling pathway in CRC cells.

In summary, we demonstrated that ILL suppressed proliferation, induced autophagy, and regulated G2/M phase cell cycle arrest in CRC cells. In addition, ILL activated the MAPK signaling pathway, which led to the induction of apoptosis and autophagy through the ROS–mediated signaling pathway (Figure 7). These results highlight the potential of ILL as a novel anticancer agent and reveal the underlying mechanisms of CRC treatment.

## 4. Material and Methods

### 4.1. Chemicals and Antibodies

Isolinderalactone was purchased from AdooQ Bioscience (Purity > 98%, Irvine, CA, USA). Anti–β–actin antibody (A5316) and other chemicals were all purchased from Sigma Chemical Co., Ltd. (St. Louis, MO, USA). The antibodies utilized in this study, including anti–cleaved caspase–9 (#7237), anti–cleaved caspase–3 (#9661), anti–Bcl–2 (#4223), anti–p21 (#2947), anti–cyclin B1 (#12231), anti–p–cdc2 (#4539), anti–cdc2 (#77055), anti–p–cdc25c (#4901), anti–cdc25c (#4688), anti–LC3B (#2775), anti–p–ERK (#4370), anti–ERK (#4695), anti–p–p38 (#4511), anti–p38 (#8690), anti–p–JNK (#4668), anti–JNK (#9258), were all purchased from Cell Signaling Technology, Inc. (Danvers, MA, USA). Bradford protein assay kit was purchased from Bio–Rad (Hercules, CA, USA). PVDF membranes were purchased from Merck Millipore (Bedford, MA, USA). The Western blot chemiluminescence reagent was purchased from Amersham Biosciences (Arlington Heights, IL, USA).

### 4.2. Cell Culture

The human colorectal cancer cell lines HCT–15 and HCT–116 were purchased from BCRC (Bioresource Collection and Research Center, Hsinchu, Taiwan). The normal human colon epithelial cell line CCD 841 CoN (a kind gift from Prof. Chia–Che Chang, National Chung Hsing University, Taichung, Taiwan). All cell lines were authenticated annually using STR analysis and tested negative for mycoplasma. Cells were cultured in appropriate medium according to the suggestions from BCRC and ATCC website. HCT–15: RPMI 1640 medium with 2 mM L–glutamine adjusted to contain 1.5 g/L sodium bicarbonate, 4.5 g/L glucose, 10 mM HEPES, and 1.0 mM sodium pyruvate, 90%; fetal bovine serum, 10%. HCT–116: 90% McCoy’s 5a medium with 1.5 mM L–glutamine and 10% fetal bovine serum. CCD 841 CoN: 90% Eagle’s Minimum Essential Medium (MEM) supplemented with 1.0 mM sodium pyruvate, 2 mM L–glutamine, and 10% fetal bovine serum. All media components were purchased from Invitrogen (Carlsbad, CA, USA).

### 4.3. MTT Assay

The cell viability was evaluated using MTT assay, as previously described [41,42]. Briefly, cells were incubated in serum–containing medium (0.5 mL in 24–well plate) and allowed to adhere for 18–24 h. Solutions were always freshly prepared by dissolving 0.1% DMSO (control) or drugs in serum–containing medium. The drug–containing medium was removed after treatment for indicated time, cells were washed with PBS, and culture medium containing 500 μg/mL MTT was added for 1 h at 37 °C. After the MTT medium was removed, 0.5 mL of DMSO was added to each well. Absorbance at 570 nm was detected by a multi–well plate reader Infinite 200 Pro Tecan^TM^ (Tecan, Mannedorf, Switzerland). The absorbance for DMSO–treated cells was considered 100%.

### 4.4. RNA Isolation and Bioinformatics

Cells were subjected to RNA extraction using RNeasy Mini Kit (Qiagen, Valencia, CA, USA) and reverse transcribed at 37 °C for 60 min using Omniscript RT Kit (Qiagen, Valencia, CA, USA) in accordance with the manufacturer’s instructions. Total RNA samples (2 μg, RIN score > 9.0) underwent processing by Beijing Genomics Institute (BGI) and were then sequenced on a Hiseq2000 for SR50, following the BGI experiment pipeline. For the identification of differentially expressed genes (DEGs), genes with an adjusted P of less than 0.05 were selected. The DEGs were subsequently subjected to gene ontology and pathway enrichment analyses using established methodologies, including DAVID and X2K Expression2Kinases, as previously described [43].

### 4.5. Western Blot Analysis

Western blot analysis was performed as previously described [41,42]. Briefly, cells were prepared using M–PER mammalian protein extraction reagent supplemented with protease inhibitor cocktail (Thermo Scientific, Rockford, IL, USA) and then centrifuged at 13,000× *g* at 4 °C for 10 min. The protein concentration in the supernatants was quantified using a Bradford Protein Assay Kit (Bio–Rad, Hercules, CA, USA). Electrophoresis was performed on a 10 or 12% SDS–PAGE gel using a Mini–PROTEAN Tetra cell electrophoresis System (BioRad, Hercules, CA, USA) with 20 μg of protein extract loaded per lane. The resolved proteins were subsequently transferred to PVDF membranes and blocked with 5% skim milk for 1 h at room temperature. Primary antibodies specific to the target proteins were then added to incubate at 4 °C overnight. Following primary antibody incubation, the PVDF membrane was washed three times with TBS/0.2% Tween–20 at room temperature and subsequently incubated with the appropriate horseradish peroxidase–conjugated secondary antibody (goat anti–mouse or anti–rabbit, 1:10,000, Sigma Chemical, St. Louis, MO, USA) for 1 h at room temperature. All protein bands of interest were detected using Western Lightning Chemiluminescence Reagent Plus (Amersham Biosciences, Arlington Heights, IL, USA).

### 4.6. DCFDA Assay

ROS were detected using 2′,7′–dichlorofluorescein diacetate (DCFDA) assay, which was performed as previously described [42]. Briefly, cells were incubated with the indicated concentrations of ILL and NAC for 24 h in 6 cm dishes. After 24 h incubation, cells were stained with 10 μM DCFDA (by adding 4 μL of DCFDA (5 mM stock in DMSO) to culture dishes) at 37 °C for 30 min. Cells were then harvested with trypsin/EDTA, collected, washed with PBS, and immediately analyzed using a BD Accuri C6 flow cytometer.

### 4.7. Monodansylcadaverine (MDC) Staining

For staining of autophagic vacuoles, cells were incubated with indicated concentration of ILL, CQ, NAC, and U0126 for 24 h in 6–well plates. The cells were washed with PBS and stained with MDC (0.05 mM in DMSO) at 37 °C for 30 min in the dark. After being washed with PBS, cells can be fixed with 4% paraformaldehyde, and images were captured using an Olympus BX41 fluorescence microscope (excitation filter: 335– nm and emission filter: 420–nm).

### 4.8. Flow Cytometric Analysis

Cell cycle analysis was conducted using flow cytometry as previously described [41]. Briefly, approximately 3 × 10^5^ cells were exposed to the indicated concentrations of ILL for 24 h. Subsequently, the cells were harvested using trypsin/EDTA, followed by PBS wahsing, and fixation with cold 100% ethanol overnight. The fixed cells were then stained with a solution containing 20 μg/mL propidium iodide, 0.2 mg/mL RNase A, and 0.1% Triton X–100 for 30 min in the dark. Flow cytometry analysis was performed using BD Accuri C6 flow cytometer, with data collection and analysis carried out using the Cflow^®^ software (1.0.264.21).

### 4.9. Annexin V–FITC Staining

The annexin V–FITC staining was performed as previously described [41]. Briefly, cells were incubated with indicated concentration of ILL for 24 h. The apoptotic cell death was examined using annexin V–FITC detection kits according to the manufacturer’s instructions (BD Biosciences, San Diego, CA, USA). The data (ten thousand events for each sample) were collected and analyzed by the BD Accuri C6 flow cytometer.

### 4.10. Transmission Electron Microscopy

CRC cells were fixed with 2.5% glutaraldehyde in 0.1 M cacodylate buffer overnight at 4 °C and postfixed in 1% osmium tetroxide in 0.1 M cacodylate buffer for 1 h. The cells were then stained with 2% uranyl acetate and subjected to gradient dehydration with ethanol/–acetone followed by embedded in Spurr’s resin. Serial ultrathin sections of 70 nm in thickness were obtained using Leica EM UC6 ultramicrotome (Leica, Heerbrugg, Switzerland). The images of sections were obtained using a Hitachi H–7500 transmission electron microscope (Tokyo, Japan).

### 4.11. Statistical Analysis

All data were shown as mean ± S.D. Statistical differences were analyzed using the Student’s *t*–test for normally distributed values and the nonparametric Mann–Whitney U–test for values with a non–normal distribution. Significant differences between groups were evaluated using analysis of variance (ANOVA) with Games––Howell test as a –post hoc test.

## Data Availability

Not applicable.

## Supplementary Materials

The following supporting information can be downloaded at https://www.mdpi.com/article/10.3390/ijms241814246/s1.
