# Supplementary material for: Isolinderalactone Induces Apoptosis, Autophagy, Cell Cycle Arrest and MAPK Activation through ROS–Mediated Signaling in Colorectal Cancer Cell Lines
Source: Int J Mol Sci. 2023 Sep 18;24(18):14246. doi: 10.3390/ijms241814246 (PMC10532319; doi:10.3390/ijms241814246)
Supplement: Supplementary file 1 [file ijms-24-14246-s001.zip › ijms-2571484-supplementary.pdf]

Figure S1

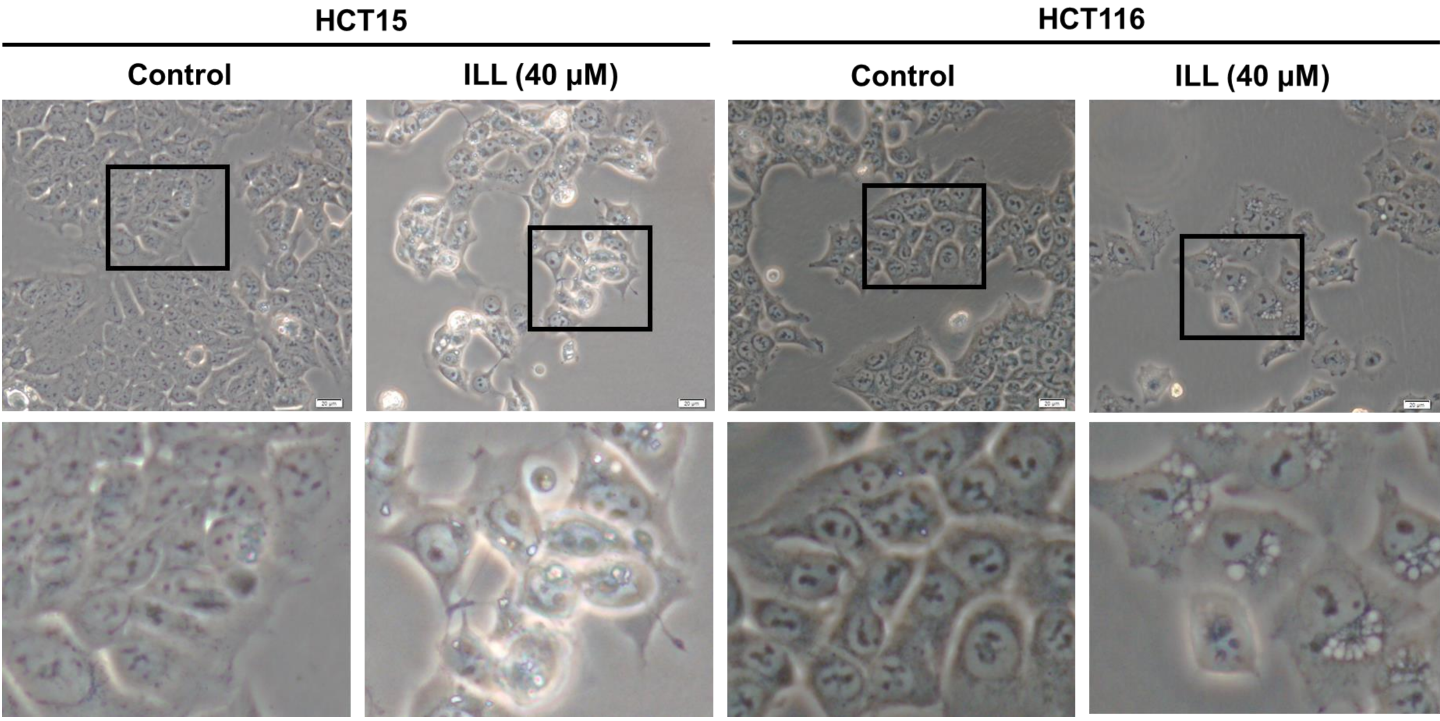

Figure S1. HCT15 and HCT116 cells were treated with 0.1 %DMSO (control) or 40 μM ILL for 24 h. Cells with vacuolation of the cytoplasm are shown.

Figure S2

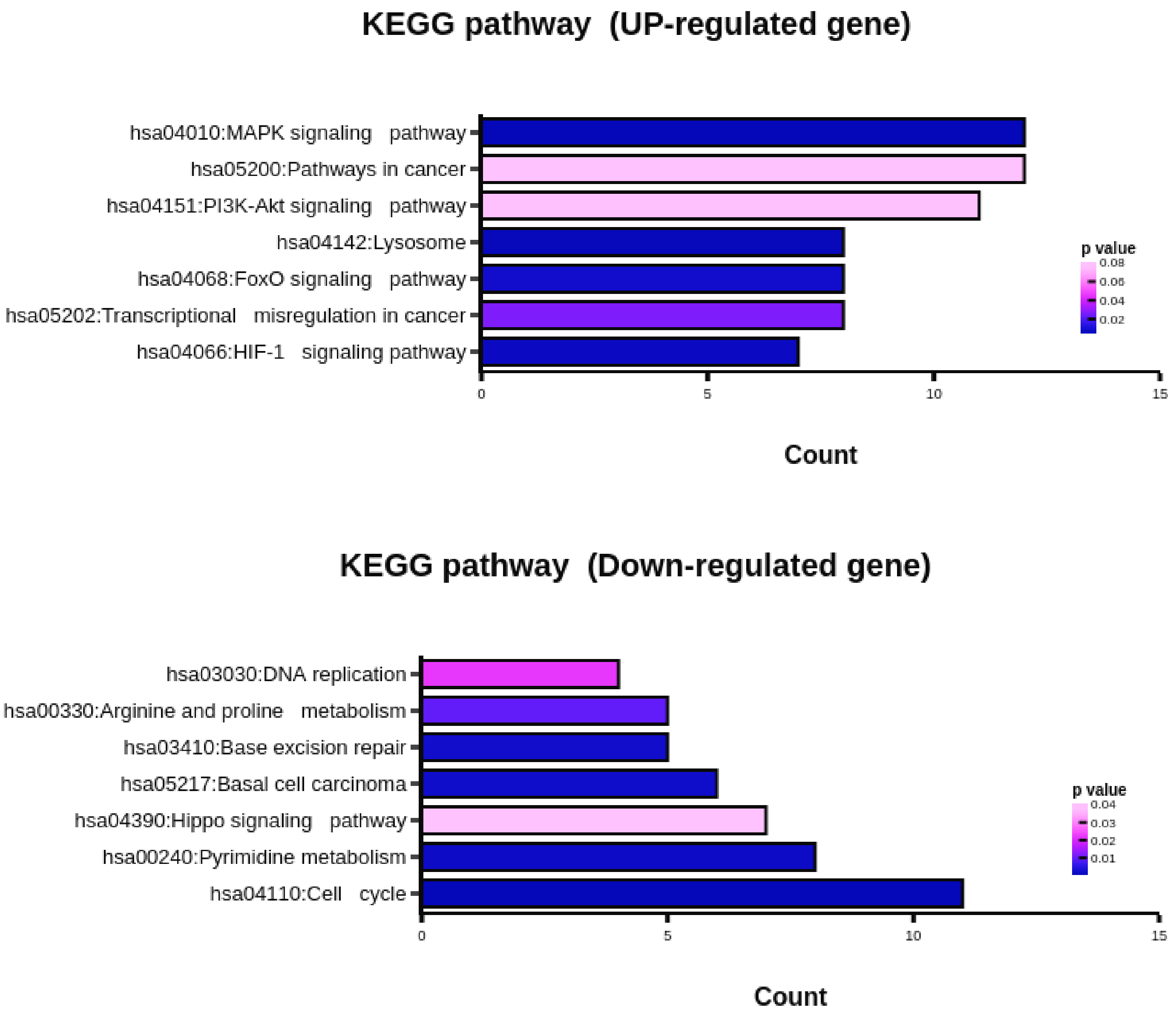

Figure S2. Cells were treated with 40 μM ILL followed by RNA-seq analysis. The KEGG pathways that were enriched after ILL-treatment are shown.

Figure S3

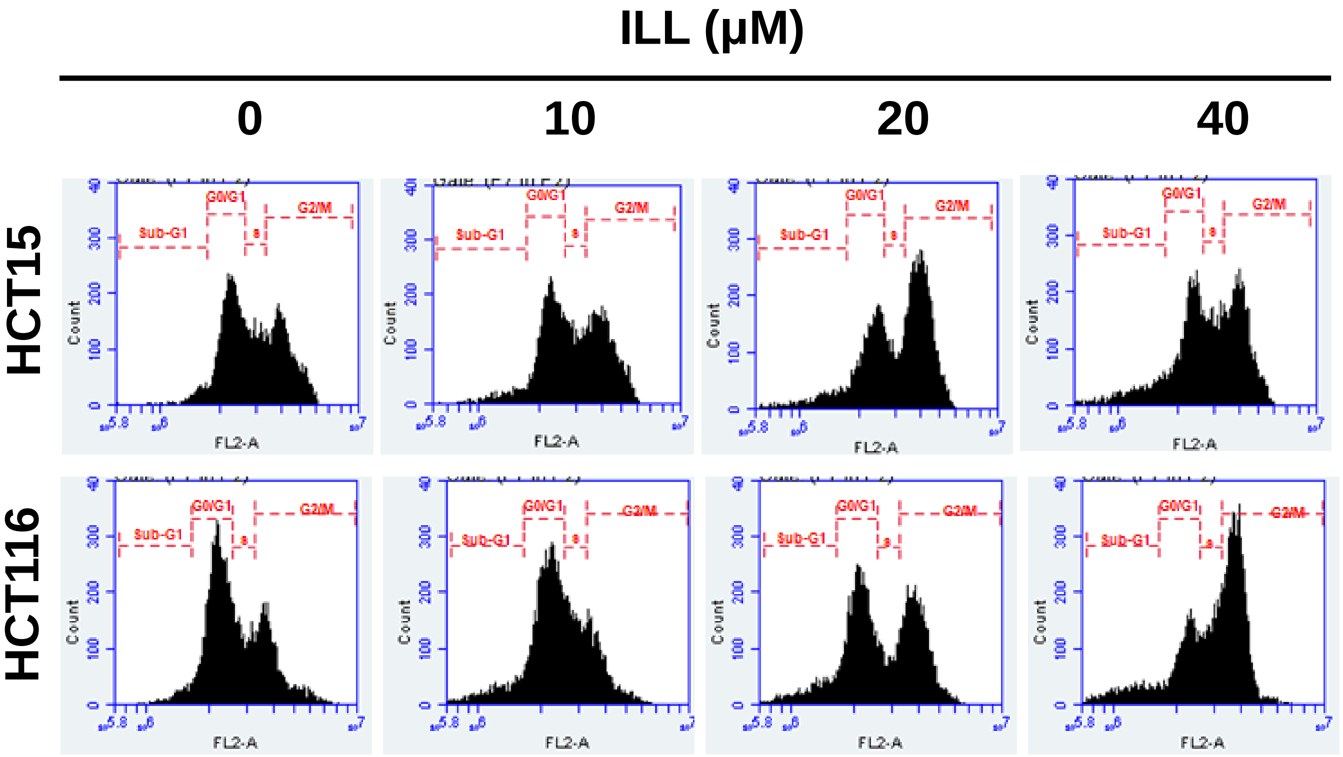

Figure S3. ILL induces G2/M phase arrest in CRC cells. Cells were treated with 0-40 μM ILL for 24 h, and the cell cycle distributions were then analyzed by flow cytometry.

Figure S4

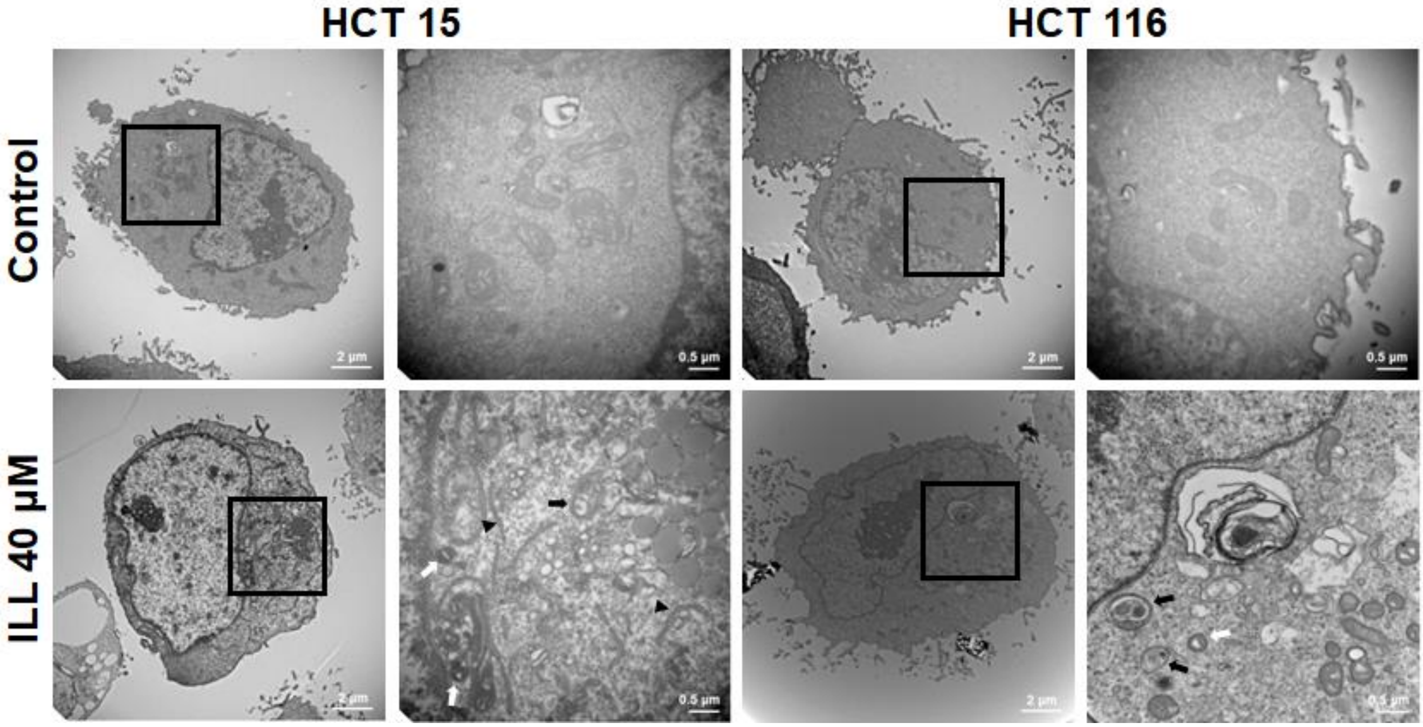

Figure S4. Effects of ILL on the activation of autophagy in CRC cells. Representative TEM micrographs were acquired from CRC cells treated with or without 40 μM ILL for 24 h. Autophagosomes (black arrow), secondary lysosome (white arrow) and phagophores (arrowhead) were more prominently observed in ILL-exposed cells than control cells.

Figure S5

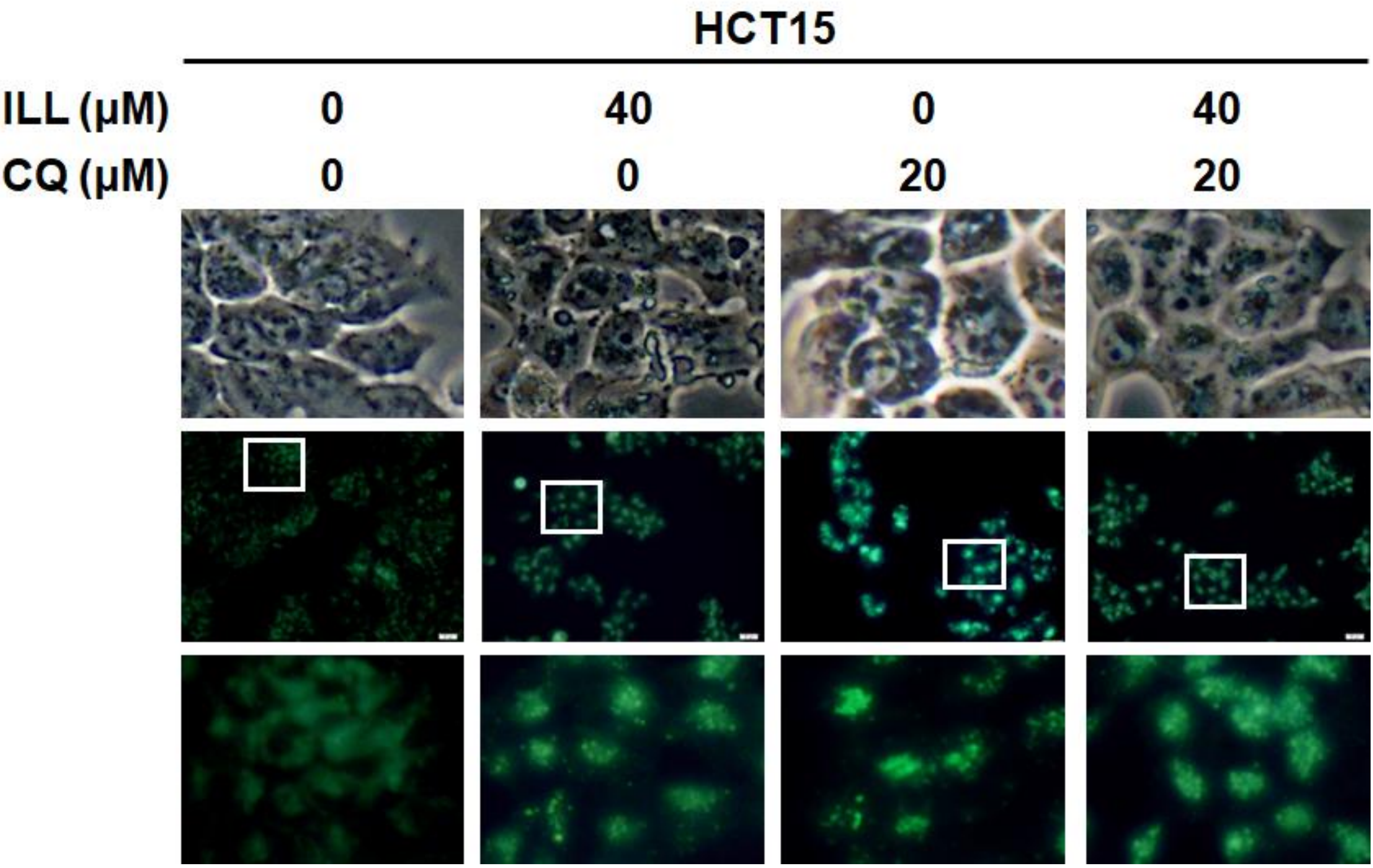

Figure S5. HCT15 cells were treated with 0.1 % DMSO (control) or 40 μM ILL in the presence or absence of the autophagy inhibitor chloroquine (CQ; 20 μM) for 24 h, and then subjected to MDC staining (green) for autophagy detection.

Figure S6

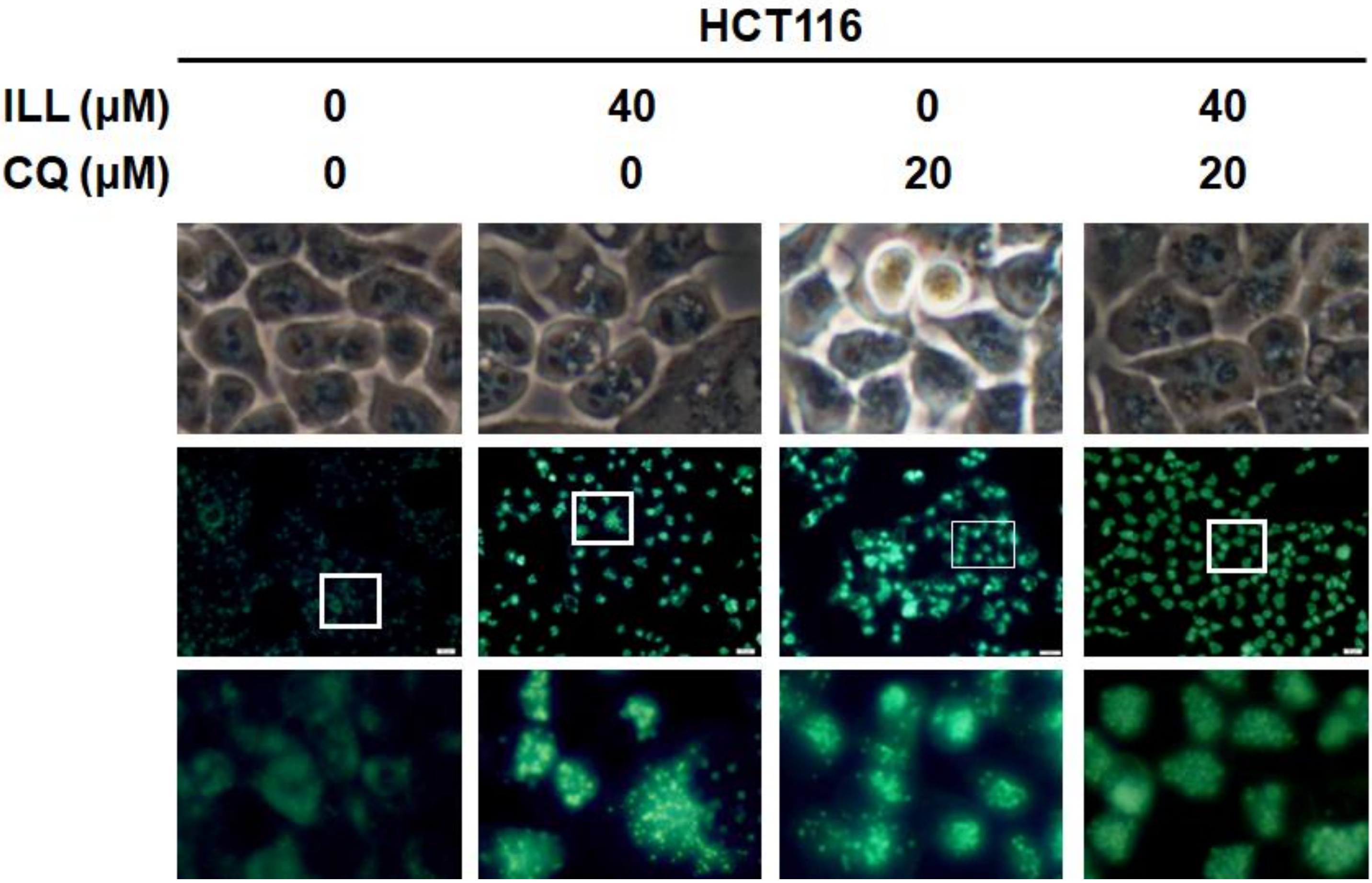

Figure S6. HCT116 cells were treated with 0.1 % DMSO (control) or 40 μM ILL in the presence or absence of the autophagy inhibitor chloroquine (CQ; 20 μM) for 24 h, and then subjected to MDC staining (green) for autophagy detection.

Figure S7

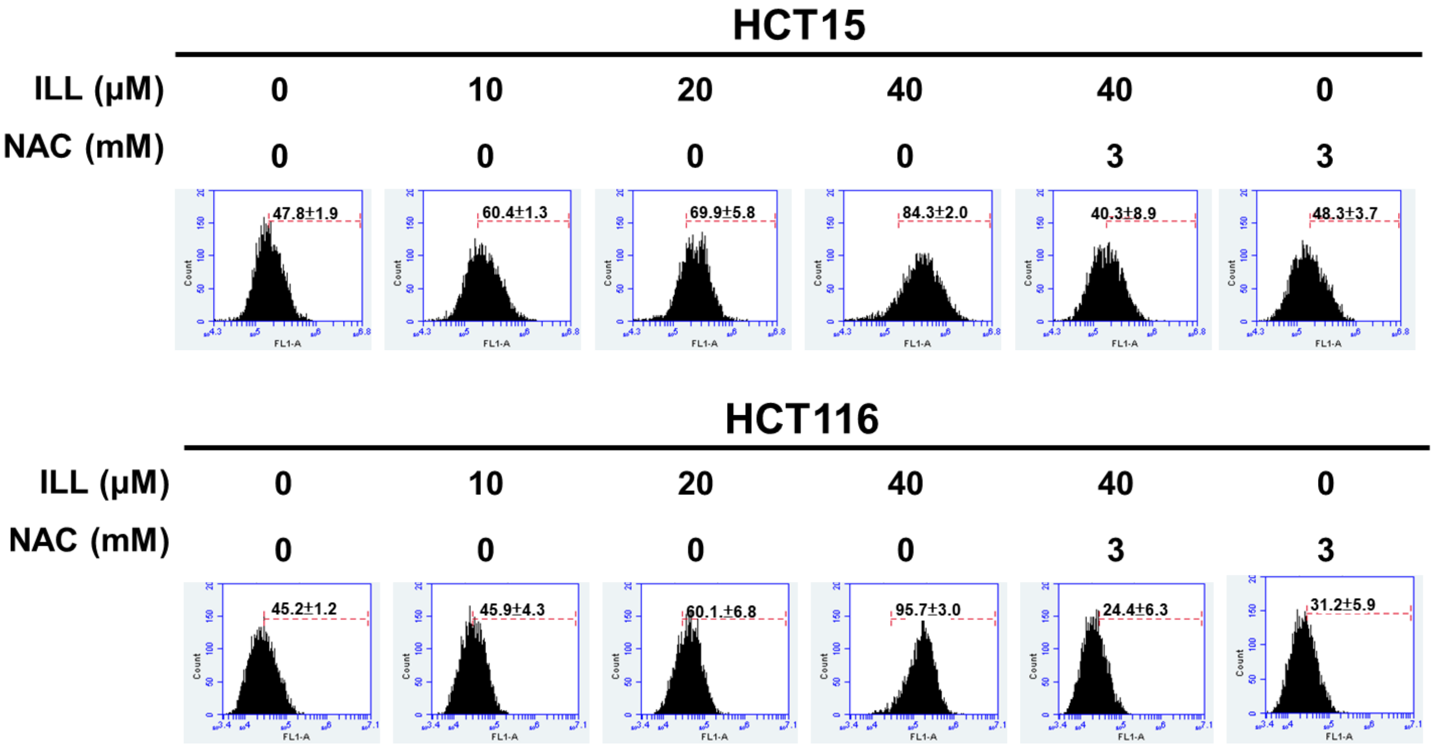

Figure S7. Effects of ILL on ROS generation in CRC cells. Cells were treated with various concentration of ILL in the presence or absence of NAC for 24h. The cells were then subjected to 10 μM DCFDA staining for 30 min, and the resulting fluorescent intensity was analyzed by flow cytometry.

Figure S8

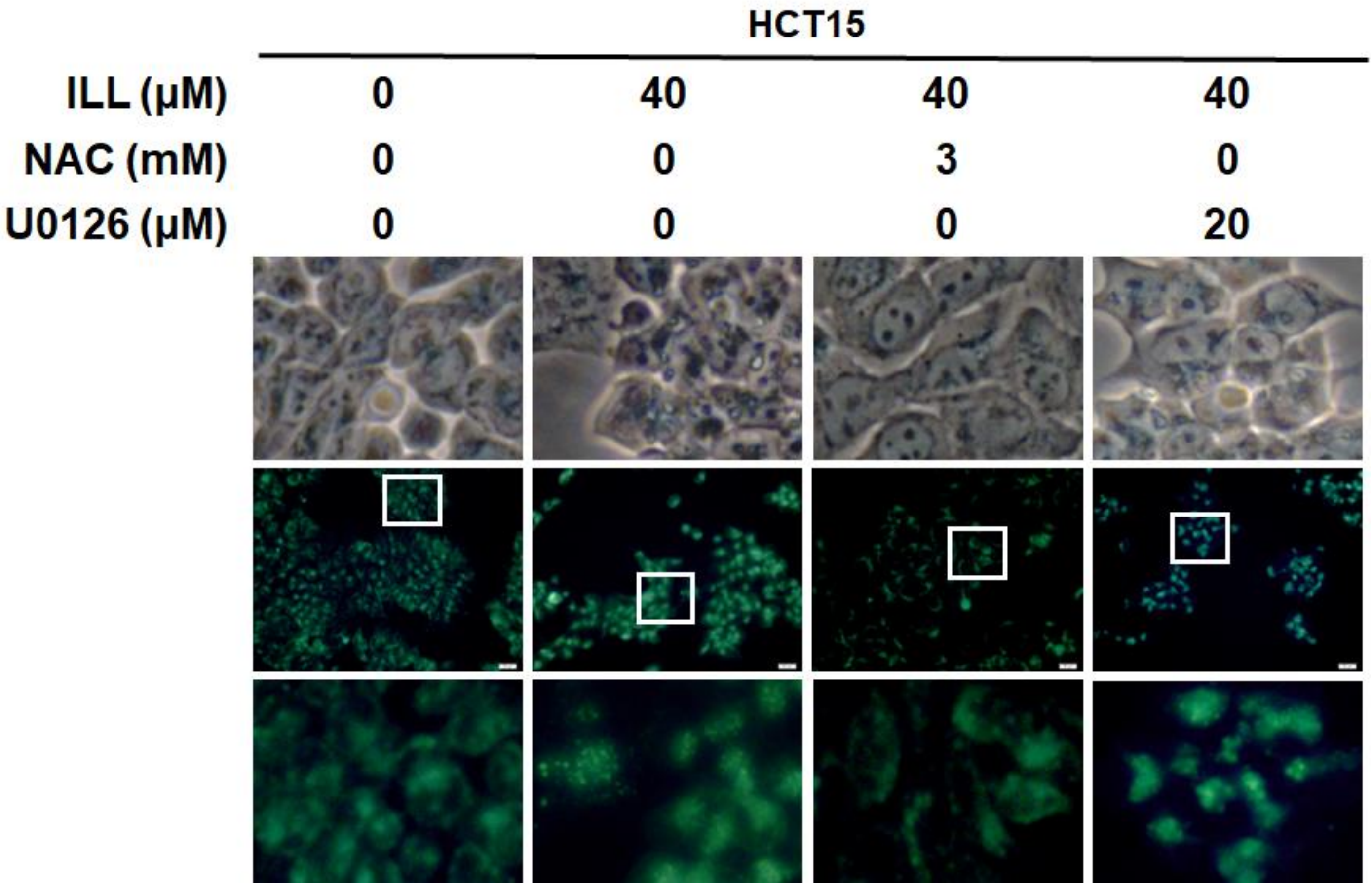

Figure S8. HCT15 cells were pretreated with 3 mM NAC or 20 μM ERK inhibitor (U0126) for 2 h, followed by treatment with 40 μM ILL for 24 h. Cells were then subjected to MDC staining and the images were captured by fluorescence microscope.

Figure S9

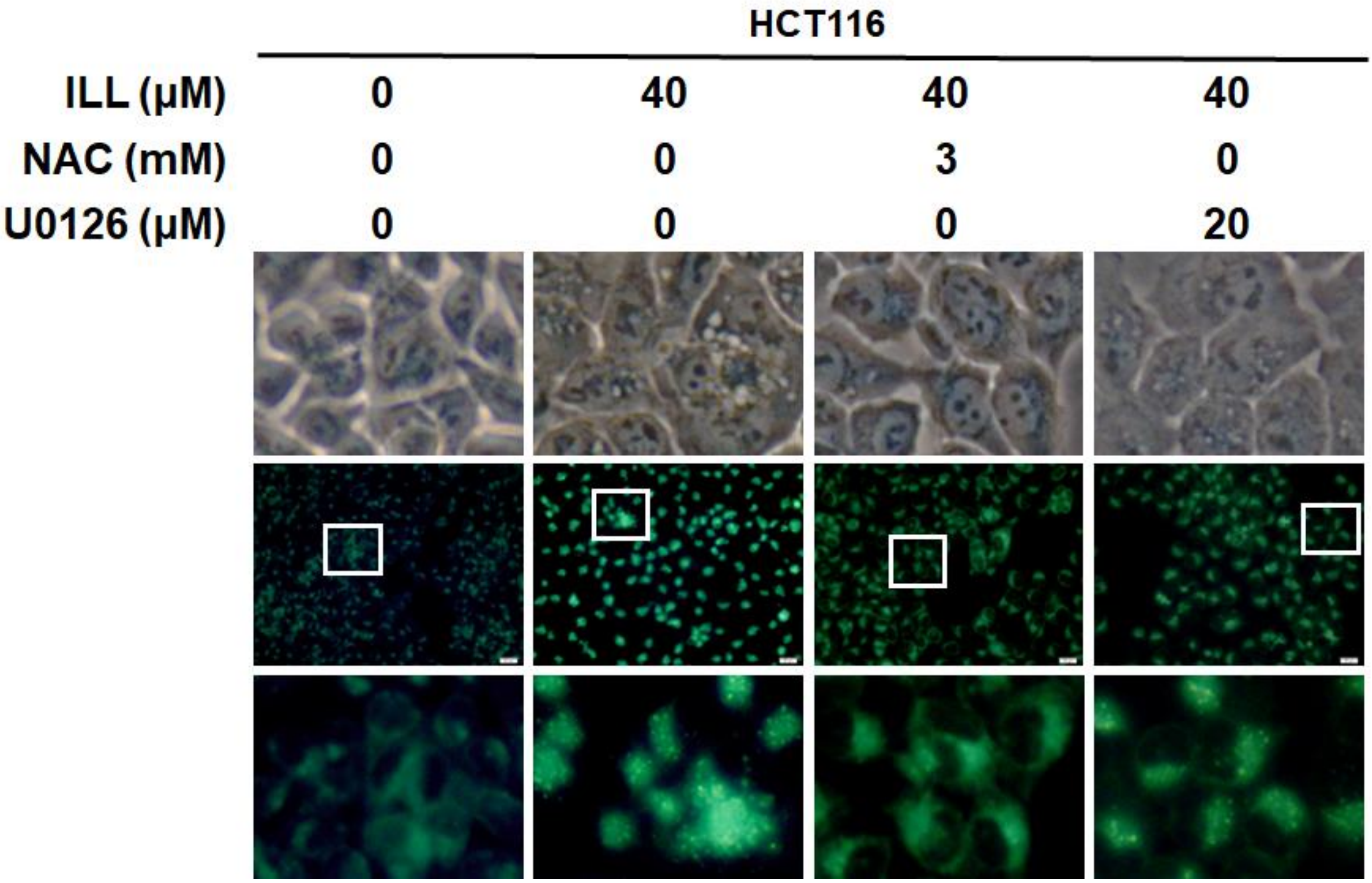

Figure S9. HCT116 cells were pretreated with 3 mM NAC or 20 μM ERK inhibitor (U0126) for 2 h, followed by treatment with 40 μM ILL for 24 h. Cells were then subjected to MDC staining and the images were captured by fluorescence microscope.
